# Genetic Diversity of *Cryptosporidium hominis* in a Bangladeshi Community as Revealed by Whole-Genome Sequencing

**DOI:** 10.1093/infdis/jiy121

**Published:** 2018-03-05

**Authors:** Carol A Gilchrist, James A Cotton, Cecelia Burkey, Tuhinur Arju, Allissia Gilmartin, Ye Lin, Emtiaz Ahmed, Kevin Steiner, Masud Alam, Shahnawaz Ahmed, Guy Robinson, Sultan Uz Zaman, Mamun Kabir, Mandy Sanders, Rachel M Chalmers, Tahmeed Ahmed, Jennie Z Ma, Rashidul Haque, Abu S G Faruque, Matthew Berriman, William A Petri

**Affiliations:** 1 Department of Medicine, University of Virginia, Charlottesville; 2 Wellcome Trust Sanger Institute, Hinxton, Cambridge, United Kingdom; 3 International Centre for Diarrhoeal Disease Research, Bangladesh; 4 Department of Statistics, University of Virginia, Charlottesville; 5 Cryptosporidium Reference Unit, Public Health Wales Microbiology, Singleton Hospital; 6 Swansea University Medical School, Singleton Park, Swansea, United Kingdom; 7 Department of Public Health Sciences, University of Virginia, Charlottesville

**Keywords:** *Cryptosporidium hominis*, genome, genotype, gp60, parasite

## Abstract

We studied the genetic diversity of *Cryptosporidium hominis* infections in slum-dwelling infants from Dhaka over a 2-year period. *Cryptosporidium hominis* infections were common during the monsoon, and were genetically diverse as measured by *gp60* genotyping and whole-genome resequencing. Recombination in the parasite was evidenced by the decay of linkage disequilibrium in the genome over <300 bp. Regions of the genome with high levels of polymorphism were also identified. Yet to be determined is if genomic diversity is responsible in part for the high rate of reinfection, seasonality, and varied clinical presentations of cryptosporidiosis in this population.

## Teaser

In the developing world, *Cryptosporidium* is one of the top causes of infant diarrhea. In a Bangladesh community we have discovered that a substantial amount of genetic recombination and genetic polymorphism in surface proteins occurs in the *Cryptosporidium hominis* parasite.

The eukaryotic protozoan *Cryptosporidium* was previously viewed as a cause of self-limited mild diarrhea and of concern only in patients with poorly controlled human immunodeficiency virus (HIV) [1]. We and others have more recently identified *Cryptosporidium* as an important diarrheal pathogen in children in low-income countries [2, 3]. The study of *Cryptosporidium* is therefore of importance as neither preventive vaccination nor infant medication is available.

The ability of the parasite to undergo asexual as well as sexual replication in the human host would be predicted to promote genetic recombination. Genetic differences between parasites could explain the high rate of reinfection, seasonality, and differences in transmissibility and clinical presentation (diarrhea vs asymptomatic infection) either independently or as part of a multifactorial etiology involving host and environmental factors [4].

We studied *Cryptosporidium hominis* infections over a 2-year period in infants in Bangladesh [5] and observed extensive parasite genetic diversity as measured by *gp60* genotyping [4, 6]. Whole-genome sequencing (WGS) of a subset of the parasites revealed high rates of sexual recombination and regions of the genome that were highly polymorphic, suggesting areas under selection.

## MATERIALS AND METHODS

### Infant Cohort

Starting in June 2014, 250 children born into an urban slum of Dhaka, Bangladesh (Section 11 of Mirpur Thana) were enrolled, in the first week after birth, into a community-based prospective cohort study of enteric infections. This neighborhood is densely populated with an average of 5.5 people living in 1.6 rooms for participants in this study. Annual median household income of participants was 12950 Taka or approximately US$158. Surveillance samples were collected monthly and from every diarrheal infection until the infants were 2 years of age [6]. Two hundred thirty-one children completed 2 years of surveillance by June 2017 and 80% of all diarrhea episodes had a stool sample analyzed for *Cryptosporidium*. Children at Mirpur had 240 *Cryptosporidium* infections (58 diarrheal; 182 subclinical), >95% of which were *C. hominis.*

### Ethical Considerations

The study was approved by the Ethical and Research Review Committees of the International Centre for Diarrhoeal Disease Research, Bangladesh (icddr,b) and by the Institutional Review Board of the University of Virginia. Informed written consent was obtained from the parents or guardians for the participation of their child in the study.

### Sampling and Specimen Testing

The diarrheal and monthly surveillance stools were tested for protozoan parasites on DNA extracted from feces by use of a multiplex quantitative polymerase chain reaction (qPCR) assay to detect the 3 parasitic protozoans *Cryptosporidium* (species), *Entamoeba histolytica*, and *Giardia lamblia* as described by Liu et al [7] but including the following modifications; the fluorophore Texas Red was used for the *Cryptosporidium* probe, 6-FAM (Fluorescein) (FAM) for *E. histolytica*, and the Minor Groove Binder (MGB), 2′-chloro-7′phenyl-1,4-dichloro-6-carboxy-fluorescein (VIC) probe for *Giardia*.

### Genotyping Assay

The polymorphic region within the *gp60* gene was used to genotype *Cryptosporidium*-positive samples (by nested PCR) using the primers and conditions previously described [8].

Sanger sequencing (GENEWIZ) was utilized to obtain *gp60* sequences. Samples were grouped as part of the same infection if they occurred within 65 days of the preceding positive sample, unless the sample was of a different *gp60* genotype. In the *gp60* genotype nomenclature used, the infecting *Cryptosporidium* species was indicated by a roman numeral (I = *C. hominis*; II = *C. parvum*). The lowercase alphabet was used to indicate the single-nucleotide polymorphism (SNP)–based allele family and the uppercase alphabet and number was used to describe the microsatellite region [8]. The genotype of an infection was deduced from the typed samples; representative sequences were submitted to GenBank (MG694234– MG694238) and are detailed in Supplementary Data 1.

### Whole-Genome Sequencing

WGS required larger volumes of stool than are routinely collected. We therefore “fast tracked” stool samples for oocyst processing by using a point-of-care assay (*Cryptosporidium/Giardia* QUIK CHEK, TechLab Inc) to test stool samples with a high risk of being infected with *Cryptosporidium* parasites in our population (diarrheal stools and stool samples from children at 9 and 11 months of age, the time of peak of both symptomatic and asymptomatic *Cryptosporidium* infection). Samples underwent initial processing within <8 days of collection. *Cryptosporidium* oocysts were purified directly from stool samples of 2 g of semisolid stool or approximately 2 mL of liquid stool material using the protocol of Hadfield et al [9]. Sixty-three of these samples resulted in sufficient numbers of oocysts to be submitted for WGS as described in the Supplementary Methods.

### Sequence Analysis

An improved WTSI reference genome assembly for *Cryptosporidium parvum* reference genome was generated using DNA purchased from American Type Culture Collection (*Cryptosporidium parvum* Tyzzer ATCC PRA-67D) (9.1Mb) and using long-read sequences (Pacific Biosciences) and Bangladesh sequence, reads were mapped and SNPs identified as described in the Supplementary Methods. In 1 infection, high-quality genomic information was collected from 2 aliquots from the same stool (icddr,b 3 and 4) and in this case the results from the read mapping were similar, indicating that only minor variation occurred in the parasite population within the same host. A total of 36780 SNPs varied between the Bangladesh *C. hominis* isolates; however, only 1582 (4.3%) occurred with a frequency >20%. A neighbor-joining tree based on pairwise distances estimated using Plink 1.90B3 was constructed using Phylip 3.69. To identify regions of highly polymorphic SNPs, the values for pi and Tajima’s D were calculated using VCFtools (v0.1.15), then an average per 1-kb window was calculated with bedtools. Estimates of *R*^2^ were also calculated done using VCFtools (version 0.1.15) for all variable sites within 2 kb of each other. All quantitative data were plotted using R version 3.3.0.

## RESULTS

### *gp60* Genotyping Data

**Figure 1.**
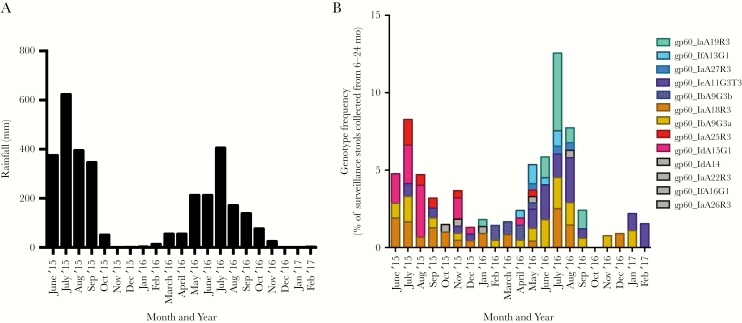
The frequency of *Cryptosporidium hominis* infections was dependent on both the time of year and the parasite genotype. Infants <6 months are infrequently infected with *Cryptosporidium* and in our study cohort none were infected. The risk of experiencing a *Cryptosporidium* infection increases thereafter. The graphs therefore, start at the beginning of the second study monsoon (2015) when *Cryptosporidium* infections began to be observed in the Mirpur cohort who were >6 months of age. The x-axis in both graphs indicates the month and year. *A*, Rainfall in the Dhaka area. The y-axis indicates total monthly rainfall (mm). *B*, The number of surveillance stools collected from children 6–24 months in age was used as a surrogate marker for the number of children participating in the study who were susceptible to cryptosporidiosis. The y-axis indicates the percentage of children experiencing genotyped *Cryptosporidium* infections occurring during this time period (surveillance and diarrheal). Genotypes are indicated by color with the exception of genotypes which occurred with a frequency of <1% in that month’s samples, which are all colored gray. Only 2 genotypes (IaA18R3 and IbA9G3a) were common in both 2015 and 2016. The months March–June 2017 were omitted from the graph as <50 participants of the correct age range remained in the study.

There were 58 diarrheal and 182 asymptomatic *Cryptosporidium* infections identified in the first 2 years of life. Genotyping at the *gp60* locus was attempted in all but 6 of the *Cryptosporidium*-positive samples. For 136 infections, subtyping of the *C. hominis* parasite was successful. Thirteen different *C. hominis* genotypes were identified in the Bangladesh cohort. The most abundant subtypes in our population were *C. hominis* IaA18R3 (19.1 %), IaA19R3 (13.2%), IaA25R3 (4.4 %), IaA27R3 (2.2%), IbA9G3a (16.9%), IbA9G3b (5.1%), IdA15G1 (11%), IeA11G3T3 (18.4%), and IfA13G1 (5.9%). The remaining genotypes were each present in <2% of all infections (IaA22R3, IaA26R3, IdA14, IfA16G1) (Figure 1). Minor differences in the previous species-specific qPCR and *gp60* genotyping assay results were resolved as described in the Supplementary Methods [6].

A weakness in the *gp60* genotyping system is that coinfections with multiple *gp60* genotypes could not be easily identified as only the genotype that constituted the majority of the parasite cells in a sample would be typed. In 4 cases, a switch in *gp60* genotype indicated that either successive *C. hominis* infections had taken place (interval between positive samples 37 ± 20 days) or a change in the frequency of coinfecting *C. hominis* genotypes occurred. In this work we defined a new infection as occurring when we identified a discordant genotype or when >2 months had elapsed from the prior positive stool sample.

*Cryptosporidium* infections were more frequent during the monsoon (early June–late September of 2015 and 2016) as has been previously described (Figure 1) [10]. Gp60 genotyping revealed that some genotypes (eg, IaA25R3 and IdA15G1) were only detected in 2015, and others only in 2016 (IaA19R3 and IfA13G1) whereas others were detected throughout the study (eg, IbA9G3 and IaA18R3) (Figure 1). We concluded that the *C. hominis* infections in this cohort were genetically diverse as judged by *gp60* genotyping. Diarrhea was present in approximately 30% of infections and was not significantly associated with any of the genotypes (data not shown). Relatively few of the infections that occurred in the same child (n = 5) were genotyped in both infections; therefore, we were not able to determine if genotype-specific immunity occurred in our population.

### Whole-Genome Resequencing

**Figure 2.**
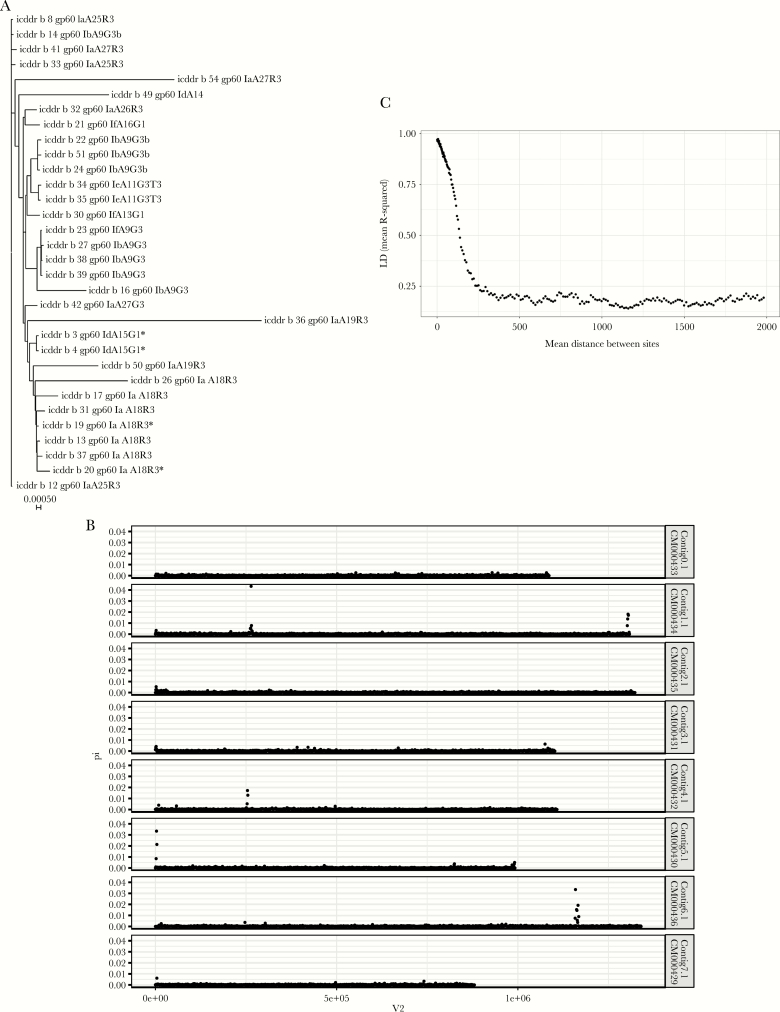
Genomic diversity of Bangladesh *Cryptosporidium hominis. A*, Phylogenetic tree of *C. hominis* genomes. Neighbor-joining tree was based on the high confidence single-nucleotide polymorphisms (SNPs) occurring with a frequency ≥0.2 in the 32 Mirpur genomes. Branches are labeled with genome ID and associated *gp60* genotype. Scale bar indicates genome-wide distance between samples in nucleotide substitutions per site. *Genomes from a “paired” oocyst preparation (isolated from different aliquots of the fecal material collected from a single child at one time point [icddr,b 3 and 4] or at a 10-day interval [icddr,b 19 and 20]), indicating that diversity can occur in the parasite population within a single child. *B*, *Cryptosporidium hominis* polymorphic regions. Graph indicates the distribution of allele frequencies, and the number of SNPs on the *C. hominis* chromosomes. Pi (y-axis) is defined as the average number of nucleotide differences per nucleotide site between 2 samples, and is a measure of allelic heterogeneity. The x-axis (V2) indicates the chromosome position (bp). *C*, High rates of recombination are apparent in this *C. hominis* population. Average linkage disequilibrium (LD) (y-axis) between neighboring SNPs as a function of the distance (bp) separating them in the genome (x-axis) for Bangladesh *C. hominis*. The graph was generated using the data from 32 Bangladesh *C. hominis* genomes, which had >80% genome coverage of at least 10 times. The pairwise values were calculated using the SNPs with minor allele frequency >0.20. The y-axis indicates the average *R*^2^ value (the square of the correlation coefficient of 2 SNPs) and x-axis the physical separation 0–2000 bp.

The Cq (quantitation cycle) is closely related to the amount of input DNA and allowed us to identify 140 high-parasite-burden samples (Cq <20) for oocyst purification. Sequencing libraries were prepared from the purified DNA of 63 isolates out of 108 high parasite burden samples (Supplementary Table 1). In 32 samples the sequences were both derived from *C. hominis* and had >80% genome coverage of 10 times (Figure 2A). Fifteen of these were diarrheal isolates and 17 were isolated from subclinical infections as defined by Steiner et al [6]. We discovered 36780 SNPs that varied within our Bangladeshi population with 4% (1582) of SNPs occurring with a frequency >0.2 in the 32 whole genomes sequenced. A slight increase in SNP density was observed in the subtelomeric DNA but, with a few exceptions (discussed later), the SNPs appeared evenly distributed throughout the genome (Figure 2B). The virulence of the isolates did not reflect genetic relatedness at a genomic level and *gp60* subgenotypes were not necessarily closely related. For instance, in the phylogenetic tree based on genomic data while independent isolates of IaA18R3 were in a single branch, the 3 IaA25R3 and 3 IaA27R3 genotypes did not group together (Figure 2A). We therefore concluded that *gp60* was not fully capturing the pattern of relatedness across the genome as a whole, likely due to frequent sexual recombination (Figure 2A) [11].

### Recombination

To examine the amount of recombination occurring within the Bangladesh parasite population, we measured the decay in linkage between SNPs as a function of their physical separation (bp). The plotted association between common SNPs with a minor allele frequency of ≥0.2 with the decay of *r*^2^ with distance was very short in *C. hominis* (<300 bp) (Figure 2C) [12]. We concluded that there was a high rate of recombination in the *C. hominis* genome, even within this limited set of resequenced genomes from a defined community.

### Highly Polymorphic Regions of the Genome

We were able to identify 7 regions of particularly high nucleotide diversity in this population (Figure 2B; Supplementary Table 2). As expected, one of these regions included the polymorphic *gp60* gene (*C. parvum* ID: cgd6_1080) and extended into the neighboring DNA, which encoded a gene of unknown function (*C. parvum* ID: cgd6_1070). Other highly diverse alleles in the Bangladesh *C. hominis* genomes include the ortholog of the highly antigenic *C. parvum* protein Cops-1 [13] and the genes encoding the insulinase-like peptidase [14]. Some of these high-diversity windows were associated with low sequencing coverage in this population, suggesting they may represent structurally variable regions, but also meaning that some samples had missing genotype calls. We concluded that there were regions of the genome likely to be under greater selective pressure for diversification—an interpretation supported by the fact that 20 of 36 1-kb windows in these regions were among the 63 windows (of 9118) with the highest value for Tajima D statistic, indicative of balancing selection increasing the number of high frequency variants. It was not possible in this limited dataset, however, to identify whether the genetic changes in these regions were associated with increased parasite virulence.

## Discussion

The diversity within *C. hominis,* even within this one community over a 2-year period, was striking. The high recombination rates resulted in *gp60* genotypes being broadly distributed across a phylogenetic tree created with WGS data, indicating the inability to assign genotypes with a single marker, no matter how polymorphic, due to recombination. Equally important was the discovery that certain regions of the genome were highly polymorphic and therefore likely under greater selective pressure to diversify, and as such potentially underlying host–parasite interactions. Polymorphic regions contained open reading frames for membrane and secreted proteins pointing to adaption of the parasite to the host and/or immune evasion, areas of potential importance in prevention and treatment.

Limitations of this study included that WGS could only be performed on *Cryptosporidium* infections present in high quantity and was only done for children 9–11 months of age. Therefore, the results of this work likely do not completely reflect the genetic diversity of *Cryptosporidium* infections in this community or in other populations [15].

In summary, this work reveals substantial genetic variation and recombination within *C. hominis.* Recognition of this complexity is an important step in the eventual control of this cause of infant morbidity and mortality.

## Supplementary Data

Supplementary materials are available at *The Journal of Infectious Diseases* online. Consisting of data provided by the authors to benefit the reader, the posted materials are not copyedited and are the sole responsibility of the authors, so questions or comments should be addressed to the corresponding author.

Supplemental Table_1

Supplemental Table 2

Supplementary data

## Notes

***Acknowledgments.*** We are grateful for the willing participation of the parents and children at the icddr,b study sites and thank the field workers, nurses, laboratory staff of the Parasitology Laboratory of icddr,b who worked for this project, without whom we could not have completed this research. Work at icddr,b is supported by the core donors (Government of the People’s Republic of Bangladesh, Global Affairs Canada, Sida, UKAid).

***Disclaimer.*** The funders had no role in study design, data collection and analysis, or decision to submit for publication.

***Financial support.*** This work was supported by the National Institute of Allergy and Infectious Diseases (grant number R01 AI-043596 to W. A. P.); the Bill & Melinda Gates Foundation (grant number OPP1100514 to A. S. G. F.); and the Wellcome Trust via their core support for the Wellcome Trust Sanger Institute (grant number 206194). K. S. is supported by the National Institutes of Health (training grant T32AI055432). TechLab Inc donated the *Giardia/Cryptosporidium* QUIK CHEKS used in this study.

***Potential conflicts of interest.*** All authors: No reported conflicts of interest. All authors have submitted the ICMJE Form for Disclosure of Potential Conflicts of Interest. Conflicts that the editors consider relevant to the content of the manuscript have been disclosed.
