# Supplementary material for: Genetic Diversity of Cryptosporidium hominis in a Bangladeshi Community as Revealed by Whole-Genome Sequencing
Source: J Infect Dis. 2018 Mar 5;218(2):259–64. doi: 10.1093/infdis/jiy121 (PMC6009673; doi:10.1093/infdis/jiy121)
Supplement: Supplemental Table 2 [file jiy121_suppl_supplemental_table_2.docx]

Supplemental Table 2

Highly Polymorphic Genes. Grey shaded rows are regions with low average coverage and high numbers of missing genotype calls.

| Chromosome/  Contig#* | | Region | ID of annotated *C. parvum* gene  (gene name)** | Paralog | Ortholog Group | Protein Characteristics | Mean  Tajima’s D | |
| --- | --- | --- | --- | --- | --- | --- | --- | --- |
| 1 | 7.1  CM000429 | 4000  -  5000 | cgd1_3850 | cgd8_20^&^ | OG5_180607 | Predicted secreted protein of *Cryptosporidium*-specific SKSR family | -0.15 | |
| 2 | 5.1  CM000430 | 2000  -  5000 | cgd2_4380  (*C*ops-1) |  | OG5_144209 | Conserved membrane protein | 1.452 | |
| 3 | 3.1  CM000431 | 1075000 –  1076000 | cgd3_4260*** | cgd3_4270 | OG5_169251 | Insulinase-like peptidase | 0.172 | |
| 4 | 4.1  CM000432 | 254000  -  256000 | New Annotation |  | OG5_127104 | Poly(A) polymerase | 0.496 |  |
| 6 | 1.1  CM000434 | 264000  -  266000 | cgd6_1080 (*gp60*)  cgd6_1070 |  | OG5_222676  OG5_128239 | Both conserved membrane proteins | 1.747 |  |
|  |  | 1302000  -  1306000 | New Annotation |  |  | Insulinase-like peptidase | 3.112 |  |
| 8 | 6.1  CM000436 | 1158000  -1169000 | cgd8_690 | cgd8_660^&^ | OG5_180678 | Potential membrane protein | 2.350 |  |

*Contig IDs from the WTSI *C. parvum* Iowa II assembly and the current reference genome (Heiges et al, 2006)

**ID from CryptoDB (Heiges et al, 2006)
