# Supplementary material for: Genetic Diversity of Cryptosporidium hominis in a Bangladeshi Community as Revealed by Whole-Genome Sequencing
Source: J Infect Dis. 2018 Mar 5;218(2):259–64. doi: 10.1093/infdis/jiy121 (PMC6009673; doi:10.1093/infdis/jiy121)
Supplement: Supplementary data [file jiy121_suppl_supplementary_data.docx]

**Supplemental Data 1**

Representative sequences of isolates from each *gp60* genotype found in this study

>MrMSP1-10C [organism=Cryptosporidium hominis] isolate IaA18R3 GP60

(GP60) gene, partial cds

TATTCTCAGCCCCGGCCGTTCCACTCAGAGGCACCTTGAAGGATGTTTCTGTTGAGGGATCATCATCATCATCATCATCATCATCATCATCATCATCATCATCATCATCATCAACATCGACCGTCGCACCAGCTCCAAAGAAAGAAAGAACTGTAGAGGGCGGCACGGAAGGAAAGAACGAAGAAAGCAGTCCAGGTTCTGAAGAACAAGACGGTGGTAAGGAAGACGGTGGTAAGGAAAACGGTGAAGGAGACACAGTAGACGGGGAACAAACCGGGAGTGGTTCTCAAGTTACTCCATCTGGAAGTGCCGGCACAGCTACCGAGTCCACAGCAACTACTACTCCAAAGGAAGAATGTGGTACTTCATTTGTCATGTGGTTCGAGAAAGGCACCCCGGTTGCGACCTTGAAGTGTGGTGATTACACTATCGTCTATGCACCTATAAAAGATCAAACAGATCCCGCACCAAGATA

>MrMSP1_10F [organism=Cryptosporidium hominis] isolate IaA19R3 GP60

(GP60) gene, partial cds

GCTGTATTCTCAGCCCCGGCCGTTCCACTCAGAGGCACCTTGAAGGATGTTTCTGTTGAGGGATCATCATCATCATCATCATCATCATCATCATCATCATCATCATCATCATCATCATCAACATCGACCGTCGCACCAGCTCCAAAGAAAGAAAGAACTGTAGAGGGCGGCACGGAAGGAAAGAACGAAGAAAGCAGTCCAGGTTCTGAAGAACAAGACGGTGGTAAGGAAGACGGTGGTAAGGAAAACGGTGAAGGAGACACAGTAGACGGGGAACAAACCGGGAGTGGTTCTCAAGTTACTCCATCTGGAAGTGCCGGCACAGCTACCGAGTCCACAGCAACTACTACTCCAAAGGAAGAATGTGGTACTTCATTTGTCATGTGGTTCGAGAAAGGCACCCCGGTTGCGACCTTGAAGTGTGGTGATTACACTATCGTCTATGCACCTATAAAAGATCAAACAGATCCCGCACCAAGATA

>MrMSP3-4B [organism=Cryptosporidium hominis] isolate IaA22R3 GP60

(GP60) gene, partial cds

TCAGCCCCGGCCGTTCCACTCAGAGGCACCTTGAAGGATGTTTCTGTTGAGGGATCATCATCATCATCATCATCATCATCATCATCATCATCATCATCATCATCATCATCATCATCATCAACATCGACCGTCGCACCAGCTCCAAAGAAAGAAAGAACTGTAGAGGGCGGCACGGAAGGAAAGAACGAAGAAAGCAGTCCAGGTTCTGAAGAACAAGACGGTGGTAAGGAAGACGGTGGTAAGGAAAACGGTGAAGGAGACACAGTAGACGGGGAACAAACCGGGAGTGGTTCTCAAGTTACTCCATCTGGAAGTGCCGGCACAGCTACCGAGTCCACAGCAACTACTACTCCAAAGGAAGAATGTGGTACTTCATTTGTCATGTGGTTCGAGAAAGGCACCCCGGTTGCGACCTTGAAGTGTGGTGATTACACTATCGTCTATGCACCTATAAAAGATCAAACAGATCCCGCACCAAGATATATCTC

>MrMSP2-7C [organism=Cryptosporidium hominis] isolate IaA25R3 GP60

(GP60) gene, partial cds

CCCGGCCGTTCCACTCAGAGGCACCTTGAAGGATGTTTCTGTTGAGGGATCATCATCATCATCATCATCATCATCATCATCATCATCATCATCATCATCATCATCATCATCATCATCATCATCAACATCGACCGTCGCACCAGCTCCAAAGAAAGAAAGAACTGTAGAGGGCGGCACGGAAGGAAAGAACGAAGAAAGCAGTCCAGGTTCTGAAGAACAAGACGGTGGTAAGGAAGACGGTGGTAAGGAAAACGGTGAAGGAGACACAGTAGACGGGGAACAAACCGGGAGTGGTTCTCAAGTTACTCCATCTGGAAGTGCCGGCACAGCTACCGAGTCCACAGCAACTACTACTCCAAAGGAAGAATGTGGTACTTCATTTGTCATGTGGTTCGAGAAAGGCACCCCGGTTGCGACCTTGAAGTGTGGTGATTACACTATCGTCTATGCACCTATAAAAGATCAAACAGATCCCGCACCAA

>MrMSP3-2B [organism=Cryptosporidium hominis] isolate_IaA26R3 GP60

(GP60) gene, partial cds

GCCCCGGCCGTTCCACTCAGAGGCACCTTGAAGGATGTTTCTGTTGAGGGATCATCATCATCATCATCATCATCATCATCATCATCATCATCATCATCATCATCATCATCATCATCATCATCATCATCAACATCGACCGTCGCACCAGCTCCAAAGAAAGAAAGAACTGTAGAGGGCGGCACGGAAGGAAAGAACGAAGAAAGCAGTCCAGGTTCTGAAGAACAAGACGGTGGTAAGGAAGACGGTGGTAAGGAAAACGGTGAAGGAGACACAGTAGACGGGGAACAAACCGGGAGTGGTTCTCAAGTTACTCCATCTGGAAGTGCCGGCACAGCTACCGAGTCCACAGCAACTACTACTCCAAAGGAAGAATGTGGTACTTCATTTGTCATGTGGTTCGAGAAAGGCACCCCGGTTGCGACCTTGAAGTGTGGTGATTACACTATCGTCTATGCACCTATAAAAGATCAAACAGATCCCGCACCAAGATATATCTC

>MrMSP1-5H [organism=Cryptosporidium hominis] isolate_IaA27R3 GP60

(GP60) gene, partial cds

GCCCCGGCCGTTCCACTCAGAGGCACCTTGAAGGATGTTTCTGTTGAGGGATCATCATCATCATCATCATCATCATCATCATCATCATCATCATCATCATCATCATCATCATCATCATCATCATCATCATCAACATCGACCGTCGCACCAGCTCCAAAGAAAGAAAGAACTGTAGAGGGCGGCACGGAAGGAAAGAACGAAGAAAGCAGTCCAGGTTCTGAAGAACAAGACGGTGGTAAGGAAGACGGTGGTAAGGAAAACGGTGAAGGAGACACAGTAGACGGGGAACAAACCGGGAGTGGTTCTCAAGTTACTCCATCTGGAAGTGCCGGCACAGCTACCGAGTCCACAGCAACTACTACTCCAAAGGAAGAATGTGGTACTTCATTTGTCATGTGGTTCGAGAAAGGCACCCCGGTTGCGACCTTGAAGTGTGGTGATTACACTATCGTCTATGCACCTATAAAAGATCAAACAGATCCCGCACCAA

>MrMSP1-3A [organism=Cryptosporidium hominis] isolate_IbA9G3a GP60

(GP60) gene, partial cds

GTATTCTCAGCCCCAGCCGTTCCACTCAGAGGCACCTTGAAAGATGTTTCTGTTGAGAGCTCATCGTCATCATCATCATCATCGTCATCATCGTCAACAACAACCCCCGCACCAGCTCCAAAGAAGGCAAGAGAAGCAGATGGCGGAGAAGAAAAGAACAATGAAGAAAGCCAAACTCCCGCTAGTCCTGGAAGTGGTGGGGTGAGTGGAGGACAAGATACTCAAGGTGGCTCCAAAGGAGACGCTGAGGAAGGCACTGAAGACAATGAACAAGCCGATGAGAGTGCTACCCAACCTTCTACCCCAGGTCAAGGCTCCGATAAAACCGAATCCACAGAAACTACTCCAAAGGAGAAGTGCGGTACTTCATTTGTTATGTGGTTCGGACAGGGTGTTCCAGTCGCAACTTTGAAGTGCGGTGACTATACTATGGTCTATGCACCAGAAAAGGACAAAACAGATCCCGCACCAAGATA

>MrInDS-3H [organism=Cryptosporidium hominis] isolate_IbA9G3b GP60

(GP60) gene, partial cds

TCCGCTGTATTCTCAGCCCCAGCCGTTCCACTCAGAGGCACCTTGAAAGATGTTTCTGTTGAGAGCTCATCGTCATCATCATCATCATCGTCATCATCGTCAACAACAACCCCCGCACCAGCTCCAAAGAAGGCAAGAGAAGCAGATGGCGGAGAAGAAAAGAACAATGAAGAAAGCCAAACTCCCGCTAGTCCTGGAAGTGGTGGGGTGAGTGGAGGACAAGATACTCAAGGTGGCTCCAAAGGAGACGCTGAGGAAGGCACTGAAGACAATGAACAAGCCGATGAGAGTGCTACCCAACCTTCTACCCCAGGTCAAGGCTCCGATAAAACCGAATCCACAGAAACTACTCCAAAGGAGAAGTGCGGTACTTCATTTGTTATGTGGTTCGGAGAGGGTGTTCCAGTCGCAACTTTGAAGTGCGGTGACTATACTATGGTCTATGCACCAGAAAAGGACAAAACAGATCCCGCACCAAGATATATCTC

>MrMSP1-8G [organism=Cryptosporidium hominis] isolate_IdA15G1 GP60

(GP60) gene, partial cds

TCCGCTGTATTCTCAGCCCCAGCCGTTCCGCTCAGAGGCACCTTGAAGGATGTTTCTGTTGAGGGCTCATCATCATCATCATCATCGTCATCATCATCATCATCATCATCATCAACGACCGTCGCACCAGCTTCAAATAAGGCAAGAACTGGAGAGGACACAGGACGAAGCGAAGGAAGTCAAGGTTCTGAAGAACACCAAGACGGAGAGGACGATAGTTCAGATTCTAGTGGAGGCAGTGTAGGAGGCACAGAGAGCGGAAGTGCAGGAGGAAAGAACGAAGAAGATAGTTCAAGTTCTGGAGGTGCTCAGGACGGCAGTGGAGGCACTGCAGAAGGCGCTACTCAGTCCGAGGCTACTGCTTCTCAAGGTGCTCCATCTCAAGGTTCTGACAAAACTACCGAGTCCACACAAACTACTCCAAAGGAAGAGTGCGGTACTTCGTTTGTAATGTGGTTCGGTGAAGGTACCCCGGTTGCGACCTTGAAGTGTGGTGGTTACACTATCGTCTATGCACCTGTAAAGGATCAAGCAAATCCCGCACCAAGATATATCTC

>MrMSP1-6A [organism=Cryptosporidium hominis] isolate_IeA11G3T3 GP60

(GP60) gene, partial cds

GCTGTATTCTCAGCCCCAGCCGTCCCACTCAGAGGCACCTTGAAGGATGTTTCTGTTGAGGGCTCATCATCATCTTCATCATCGTCTTCATCTTCATCATCATCATCGTCGTCAACAACCCCAGCACCAGCTTCAAAGAAGGTAAGAGAAGCAGAAGGCAGTGAAGAAAAGGACAGCGAAGAAAAGGACAGTGAAGAAAAGGGCAGTGAAGAAGGTAGCCAAACTCCCGCTAGTCCTGGAGGTGGAGGGGTGAGTGAAGGAGATACTCAAGGTGACTCTAAAGGAGACGGAGTTAGTTCAGATGAGAACCAAAGTCAAGGTGGGGACGCTACTCCCGGATCTAGCACCCAAACTCAAGCTACTGAAAAAGAACCCGGATCTTCAGAAGCTACTCCAAAGGAAGAGTGCGGTACTTCATTTGTAATGTGGTTCGGACAGGGTGTTCCAGTTGTAACTTTGAAGTGTGGTGGTTATACTATGGTCTATGCACCAGAAAATGGCAAAACAGATCCCGCACCAAGATATAT

>MrInMs-1F [organism=Cryptosporidium hominis] isolate_IfA13G1 GP60

(GP60) gene, partial cds

GCTGTATTCTCAGCCCCAGCCGTTCCACTCAGAGGCACCTTGAAAGATGTTTCTGTTGAGAGCTCATCATCATCATCATCATCATCATCATCATCATCATCGTCAACAACAACCCCCGCACCAGCTCCAAAGAAGGCAAGAGAAGCAGAAGGCAAAGAAGCAGAAGGCAAAGAAGAAGAGGGCAGTGAAGAAAGCCAAGGTCCCACTAGTTCTGGAAGTGGAGTGGGGAGTGAAGGAAATGATCAAGGTGACTCTAAAGGAGACGGAGCTAGTGAAGATGATAATAAAAATCAAGATGGTGACACTTCTTCCGAATCTGTCACCCCAACTCAAGCTACTCCAAAGGAAGAATGTGGTACTTCATTCATAATGTGGTTCGGAGAAGGTACTCCAGCCACAACTTTGAAGTGTGGTGGTTACACTATCGTCTATGCACCAGAAAAGAACAATAGAGAACCCGCACCA

>MrMSP3-1A [organism=Cryptosporidium parvum] isolate IIcA5G3a GP60

(GP60) gene, partial cds

GTCCGCTGTATTCTCAGCCCCACCCGTTCCACTCAGAGGCACTTTAAAGGATGTTTCTGTTGAGAGCTCATCGTCATCATCGTCATCGTCAACAACAACCCCCGCACCAGCTCCAAAGAAGGTAAGAGAAAGCGAAGAAGGGAAGAACAGTGAAGATAGTCAAACTCCCGCTAGTCCTGGAAGTGATTCTCAGGATAGCTCTAAAGGAGACGAAGTTGTAGGTGGAGGCGCTTCCGGATCTAGTACCCCAACTCAAGCTGCTGAAAAGGAGCCCGAAACTCCAGAATCTACTCCAAAGGAAGAATGTGGTACTTCATTTATAATGTGGTTCGGAGAAGGTACTCCAGCCACAACTTTGAAGTGCGGTGGCTACACTATCGTCTATGCACCAGAAAAGGATAATAAAGAACCCGCACCAAGATATATCTC

>MrMSP3-3F [organism=Cryptosporidium parvum] isolate IIdA13G1 GP60

(GP60) gene, partial cds

GTCCGCTGTATTCTCAGCCCCAGCCGTTCCACTCAGAGGCACTTTAAAGGATGTTTCTGTTGAGGGTTCATCATCATCATCATCATCATCATCATCGTCATCATCATCATCATCAACATCGACTGTAGCACCAACTCCAAAGAAAGAAAGAACTGGAGAGGAAGTAGGTAATCCAGGTTCTGAAGGTCAGGACGGTAAAGGAGACACTGAAGAAACAGAAGACAATCAGACCGAGAGTACTGTTTCTCAAAATACTCCAGCTCAAACTGAAGGCACAACTACCGAAACCACAGAAGCTGCTCCAAAGAAAGAGTGCGGTACTTCATTTGTTATGTGGTTCGGAGAGGGTGTTCCAGTTGCATCTTTGAAGTGTGGCGACTATACTATGGTCTATGCACCAGAAAAGGACAAAACAGATCCCGCACCAAGATATATCTC

>MrMSP2-6E [organism=Cryptosporidium hominis] isolate IdA14 GP60

(GP60) gene, partial cds

TCCGCTGTATTCTCAGCCCCAGCCGTTCCGCTCAGAGGCACCTTGAAGGATGTTTCTGTTGAGGGCTCATCATCATCATCATCATCATCATCATCATCATCATCATCAACGACCGTCGCACCAGCTTCAAATAAGGCAAGAACTGGAGAGGACACAGGACGAAGCGAAGGAAGTCAAGGTTCTGAAGAACACCAAGACGGAGAGGACGATAGTTCAGATTCTAGTGGAGGCAGTGTAGGAGGCACAGAGAGCGGAAGTGCAGGAGGAAAGAACGAAGAAGATAGTTCAAGTTCTGGAGGTGCTCAGGACGGCAGTGGAGGCACTGCAGAAGGCGCTACTCAGTCCGAGGCTACTGCTTCTCAAGGTGCTCCATCTCAAGGTTCTGACAAAACTACCGAGTCCACACAAACTACTCCAAAGGAAGAGTGCGGTACTTCGTTTGTAATGTGGTTCGGTGAAGGTACCCCGGTTGCGACCTTGAAGTGTGGTGGTTACACTATCGTCTATGCACCTGTAAAGGATCAAGCAAATCCCGCACCAAGATATATCTC

>MrMSP1-7F [organism=Cryptosporidium hominis] isolate IfA16G1 GP60

(GP60) gene, partial cds

TCCGCTGTATTCTCAGCCCCAGCCGTTCCACTCAGAGGCACCTTGAAAGATGTTTCTGTTGAGAGCTCATCATCATCATCATCATCATCATCATCATCATCATCATCATCATCGTCAACAACAACCCCCGCACCAGCTCCAAAGAAGGCAAGAGAAGCAGAAGGCAAAGAAGCAGAAGGCAAAGAAGCAGAAGGCAAAGAAGAAGAGGGCAGTGAAGAAAGCCAAGGTCCCACTAGTTCTGGAAGTGGAGTGGGGAGTGAAGGAAATGATCAAGGTGACTCTAAAGGAGACGGAGCTAGTGAAGATGATAATAAAAATCAAGATGGTGACACTTCTTCCGAATCTGTCACCCCAACTCAAGCTACTCCAAAGGAAGAATGTGGTACTTCATTCATAATGTGGTTCGGAGAAGGTACTCCAGCCACAACTTTGAAGTGTGGTGGTTACACTATCGTCTATGCACCAGAAAAGAACAATAGAGAACCCGCACCAAGATATAT
